# Understanding how a community-based intervention for people with spinal cord injury in Bangladesh was delivered as part of a randomised controlled trial: a process evaluation

**DOI:** 10.1038/s41393-020-0495-6

**Published:** 2020-06-15

**Authors:** Hueiming Liu, Mohammad Sohrab Hossain, Md. Shofiqul Islam, Md. Akhlasur Rahman, Punam D. Costa, Robert D. Herbert, Stephen Jan, Ian D. Cameron, Stephen Muldoon, Harvinder S. Chhabra, Richard I. Lindley, Fin Biering-Sorensen, Stanley Ducharme, Valerie Taylor, Lisa A. Harvey

**Affiliations:** 1 George Institute for Global Health, Sydney, NSW Australia; 2 John Walsh Centre for Rehabilitation Research, Kolling Institute, Sydney Medical School/Northern, University of Sydney, St Leonards, NSW Australia; 3 Centre for the Rehabilitation of the Paralysed, Savar, Dhaka, Bangladesh; 4 Neuroscience Research Australia, Barker Street, Randwick, NSW Australia; 5 Muldoon Rehabilitation, 72 Liscreevin Road, Lisnarick, Co Fermanagh, BT, Northern Ireland Northern Ireland; 6 Indian Spinal Injuries Centre, Sector C, Vasant Kunj, New Delhi, India; 7 Westmead Applied Research Centre, University of Sydney, Sydney, NSW Australia; 8 Clinic for Spinal Cord Injuries, Rigshospitalet, University of Copenhagen, Copenhagen, Denmark; 9 Boston Medical Centre and Boston University School of Medicine, 725 Albany street, Boston, MA USA

**Keywords:** Neurological disorders, Neurology

## Abstract

**Design:**

Mixed methods study

**Setting:**

Community, Bangladesh

**Objectives:**

To understand how a community-based intervention for people with spinal cord injury (SCI) in Bangladesh was delivered as part of a randomised controlled trial and to gauge the perceptions of participants and healthcare professionals to the intervention.

**Methods:**

A community-based intervention was administered to 204 participants as part of a large randomised controlled trial (called the CIVIC trial). Case-managers followed-up participants with regular telephone calls and home visits over the first 2 years after discharge. The following data were collected alongside the trial: (i) chart audit of telephone calls and home visits (ii) recordings of 20 telephone calls (iii) interviews with 14 Intervention participants and four healthcare professionals including three case-managers.

**Results:**

Participants received the target number of telephone calls and home visits. Pressure injuries were identified as a problem during at least one telephone call by 43% of participants. Participants and case-managers valued regular telephone calls and home visits, and believed that calls and visits prevented complications and alleviated social isolation. Participants trusted case-managers and were confident in the care and advice provided. Case-managers expressed concerns that people with SCI in Bangladesh face many problems impacting on well-being and motivation stemming from poverty, limited employment opportunities, societal attitudes and inaccessible environments.

**Conclusion:**

A community-based intervention involving regular telephone calls and home visits was administered as intended and was well received by the recipients of the care. Nonetheless, people with SCI in Bangladesh face economic and social problems which cannot be fully addressed by this type of intervention alone.

## Introduction

People with spinal cord injury (SCI) in low- and middle-income countries (LMICs) face many challenges when discharged home after their initial injuries. Our recent cohort study of patients discharged from a large hospital in Bangladesh with recent SCI indicated that 20% of those who were wheelchair dependent at discharge had died within 2 years [1] and 32% had died within 5 years [2]. Those who had not died faced ongoing problems with pressure injuries, social isolation, lack of employment, poverty and depression [3]. Similar findings have been reported in other LMICs [4–7]. So clearly there is an urgent need to support people with SCI in the community particularly in the first few years post discharge.

In high-income countries, people with SCI are supported after discharge by developed healthcare systems. Typically, people with SCI can return to hospital for regular check-ups, and mobile teams of healthcare professionals can visit and support people with SCI in their homes [8]. Ongoing physiotherapy, counselling and other services may be provided. In addition, people with SCI often have access to sophisticated treatments and rehospitalisation if they develop complications [9–13]. However, such systems of comprehensive care post discharge are not feasible in most LMICs because they are expensive and there is a shortage of healthcare professionals and hospitals with expertise in SCI. In addition, it is difficult for people to get admitted to a hospital if they develop complications because of bed shortages and because often people have to travel long distances to hospitals on poor roads [8].

Our team developed a model of care to support people with SCI after discharge in the community (see Supplementary File 1). It was designed to be affordable and sustainable in Bangladesh. It involves regular telephone calls and home visits over 2 years by specially trained health-care professionals who act as case-managers. Initially an advertisement was placed for any healthcare professionals including nurses for the positions of case-managers but only physiotherapists applied. They were therefore used as the case-managers and reflect the likely workforce for the future if this model of care is effective. At each point of contact between the case-managers and people with SCI, the case-managers screened participants for complications (using a standardised checklist; see Supplementary File 2) and provided ongoing education, psychological support and advice. In addition, each participant was allocated $AU80 for essential goods and services, and provided with an illustrated book that provided guidance on key issues likely to be experienced post discharge (the illustrations catered for participants with limited literacy).

Our model of care (including telephone calls, home visits, $AU80 and an illustrated book) was adapted from evidence-based models used in high-income countries; and drew from evidence about the effectiveness of community-based rehabilitation in LMICs [14] and telephone-based support for people with SCI and other types of disabilities in high-income countries [15, 16]. It was also based on a study where we demonstrated the feasibility of providing advice over the telephone for people with pressure injuries in a clinical trial involving 120 people from India and Bangladesh [17]. We also conducted a pilot study, which found that this model of care could be feasibly delivered [18] by the Centre for the Rehabilitation of the Paralysed in Bangladesh (CRP): a hospital which largely serves the poor and disadvantaged with most coming from rural areas in Bangladesh and working as labourers prior to injury [19]. The full intervention is being tested in a large definitive randomised controlled trial of 410 people with recent SCI (the CIVIC trial [20]). Data collection were completed in February 2020 and are currently being analysed.

The full protocol for the process evaluation has been described in-depth elsewhere [21]. It is based on the UK Medical Research Council guidance [22] and outlines the plan to combine qualitative and quantitative data for an in-depth description of what happened on the ground. The two key components of the framework underpinning the process evaluation address many questions related to how the intervention was delivered (implementation) and how the intervention may or may not have achieved its effect (mechanism of action) taking into account the Bangladeshi situation (context). In this paper, we specifically address the first set of issues around implementation as per the protocol, namely:

- was the intervention delivered as intended?
- what was the nature of the support and advice provided by the case-managers?
- what type of issues were identified during each interaction between case-managers and participants?

Hence, the aim of the study was to understand how our community-based intervention was administered ‘on the ground’ and gauge the perceptions of the participants and healthcare professionals to the intervention.

## Methods

### Trial design

The CIVIC trial is an assessor blinded randomised control trial in which 410 people who were wheelchair dependent and about to be discharged from CRP in Bangladesh following recent SCI, are randomised to either usual care (control, *n* = 206) or usual care plus our model of care (intervention, *n* = 204) for 2 years after discharge. The primary outcome is mortality at 2 years and secondary outcomes are pressure injuries, other SCI complications (such as urinary tract and respiratory infections, depression), quality of life and participation. The trial was prospectively registered (ACTRN 12615000630516, Universal Trial Number U1111‐1171‐1876)) and the protocols for the study [20], statistical analysis [23] and process evaluation [21] have been published. Ethical approval for all aspects of the trial including the data presented in this paper was attained from the ethics committees of the CRP, Bangladesh and the University of Sydney, Australia. The CIVIC trial commenced in July 2015 and the last participant was randomised in March 2018. Data collection was completed in February 2020 but the effectiveness analyses have not yet been completed or reported, and all but two authors remain blinded.

### Setting

All participants (control and intervention) of the CIVIC trial received standard inpatient rehabilitation at CRP prior to discharge (and randomisation) and usual care after discharge. Inpatient rehabilitation included training in mobility, bladder and bowel care, vocational training, and guidance on ways to find employment and be independent at home and in the community. Usual care after discharge was variable but data collected as part of the CIVIC trial indicated that all participants (control and intervention) had a median (interquartile range) of 2 (1–5) telephone interactions with staff from CRP, and were visited by CRP staff a median (IQR) of 1 (0–2) times over the first 2 years following discharge. Participants were not routinely brought back to CRP for an outpatient follow-up after discharge and readmission for management of problems was rare because CRP had limited bed capacity and participants could not readily travel to CRP.

### Data collection for the process evaluation

#### Quantitative data

Three sources of quantitative data were collected.

**Source 1: The duration and number of telephone calls and home visits made to the 204 Intervention participants**. These data were derived from the trial records: trial staff recorded the date and duration of each telephone call and home visit (see Supplementary File 2).

**Source 2: The number and types of problems identified during the telephone calls and home visits to the 204 Intervention participants**. These data were derived from the trial records: trial staff used a checklist to record these data (see Supplementary File 2).

**Source 3: The types of equipment and services purchased for the 204 Intervention participants**. Each participant was allocated $AU80 for miscellaneous items as required. Details records were kept on how this money was spent.

#### Qualitative data

Three sources of qualitative data were collected.

**Source 1: Recordings of 20 routine telephone calls provided by the case-managers to Intervention participants**: The telephone calls were randomly selected from all Intervention participants on the trial at the time of data collection (December 2018) stratified by tetraplegia vs. paraplegia, many health problems vs. micnimal health problems (as categorised by trial staff based on their chart records), and discharged in the preceding year vs. discharged more than 1 year prior. The telephone calls were conducted by the case-managers in Bangla and were later translated and transcribed into English by a team of bilingual physiotherapists.

**Source 2: Interviews with healthcare professionals:** MSH, LAH and HL interviewed three (of six) case-managers responsible for providing the intervention to participants, and HL interviewed another healthcare professional involved in the management of the trial between December 2018 and March 2019. Another 12 healthcare providers and stakeholders from CRP and other parts of the country, including policy makers and service providers were also interviewed but their data are not reported in this paper. The interviews were semi-structured using an interview guide and were conducted in English (see Supplementary File 3a). The healthcare professionals were asked to reflect on issues such as: *What were the main issues discussed during the telephone calls and home visits? What were some of the biggest problems people with SCI face after discharge? Did they think people with SCI did/would value regular contact with healthcare professionals after discharge?*

**Source 3: Interviews with Intervention participants**: One of the authors (MSH) interviewed 14 participants from the Intervention group in their homes between December 2018 and March 2019 (6 participants from the Control group were also interviewed but their data are not reported in this paper). The person conducting the interviews knew that the participants were in the Intervention group. All Intervention participants had either completed the trial or were currently on the trial and were categorised by trial staff as tetraplegia vs. paraplegia, many health problems vs. minimal health problems (based on participants’ chart records), and discharged in the preceding year vs. discharged more than 1 year prior. Fourteen were then randomly selected to ensure representation from each category although subsequently three randomly selected participants who lived in a very remote part of Bangladesh were replaced with another three participants who lived closer to CRP to minimise travel. Participants were initially telephoned, invited to participate (none declined), and then interviewed in person in their homes. The interviews were semi-structured using an interview guide (see Supplementary File 3b). The participants were asked to reflect on the following issues: *Did they value the regular contact with the case-managers? Did the intervention help them and if so how? What were some of the biggest problems they faced post discharge?* The interviews were conducted in Bangla and later translated and transcribed into English by a team of bilingual physiotherapists.

### Analysis

Recordings of the telephone calls and interviews were analysed by the authors (LAH, MSH and HL), who have backgrounds in physiotherapy, public health and medicine, and varied experience in qualitative research. The data were managed with NVivo version 11. The authors read transcripts of telephone calls and interviews, and coded the transcripts line-by-line. Four transcripts were coded together after an in-depth discussion to better understand the background of the telephone calls and interviews, and to clarify any issues related to local context and culture. Analysis was both deductive and inductive using an overarching framework with major nodes of context, mechanisms, implementation and outcomes (as per the protocol for the process evaluation [21]), and iterative analysis of the data was conducted to form new codes. After making changes to the coding framework (Supplementary File 4), and analysing seven more transcripts together, the remaining transcripts were divided and coded separately. Weekly meetings were held over a 4-month period to discuss the analysis and clarify concepts. For this paper, all data coded to the tree node ‘mechanisms’ of the CIVIC intervention were re-analysed using a constant comparison approach of different perspectives [24].

The quantitative data were collated and tabulated. Key themes were triangulated with descriptive statistics obtained from the quantitative data to provide a better understanding of how the model of care was administered ‘on the ground’ and gauge the perceptions of the participants and healthcare professionals to the intervention.

## Results

A median (IQR) of 38 (36–40) telephone calls lasting a median (IQR) of 9.8 min (8.6–11.1), and a median (IQR) of 3 (3–3) home visits lasting a median (IQR) of 2.0 h (1.9–2.2) were provided to the 204 Intervention participants. The characteristics of the 14 Intervention participants and four healthcare professionals who were interviewed, and the 20 Intervention participants whose telephone conversations with case-managers were recorded, are shown in Table 1. Our findings are organised into key themes with the supporting quantitative findings (details in Table 2 and Supplementary files) embedded within the main text. Additional illustrative quotes are provided in Supplementary File 5.

**Table 1:** Characteristics of Intervention participants whose telephone calls with their case-managers were recorded and characteristics of the Intervention participants and healthcare professionals who were interviewed.

	Intervention participants whose telephone calls were recorded (*n* = 20)	Intervention participants who were interviewed (*n* = 14)	Healthcare professionals who were interviewed (*n* = 4)
Age, years	35 (26–44)	39 (29–47)	34 (29–40)
Gender, M:F, *n*	18:2	11:3	3:1
Years of experience, years	–	–	11 (5–16)
Time since injury, years	2.2 (1.7–2.6)	2.6 (2.2–3.4)	–
Time since discharge, years	1.5 (1.2–2.0)	2.1 (1.6–3.0)	–
Tetra vs. para, *n*	9:11	9:5	–
Minimal vs. many problems, *n*	12:8	13:1	–

All data are counts, except data for age and time variables which are reported as medians (interquartile ranges).

**Table 2:** Summary of the chart audits of the Interactions between case-managers and Intervention participants (*n* = 204) during the telephone calls and home visits.

	Telephone calls	Home visits
Acute illness/fever	51%	7%
Skin	43%	30%
Bladder	36%	14%
Depression	34%	16%
Burning sensation/pain	29%	12%
Sleep, appetite or mood	28%	16%
Bowel	26%	16%
Spasticity	23%	11%
Pain	18%	9%
Swelling	17%	12%
Urinary tract infections	16%	3%
Miscellaneous	12%	7%
Autonomic dysreflexia	5%	5%

The most common “miscellaneous” problems were those related to sexual and respiratory function, hypotension and visits to traditional healers.

Data include the number (%) of participants who reported experiencing the following problems on at least one telephone call or one home visit.

### Theme 1: Prevention and management of pressure injuries was a major focus of telephone calls between the case-managers and intervention participants

It was apparent that pressure injuries were a major focus of the telephone calls between the case-managers and Intervention participants (See Table 2). Pressure injuries and bladder-related issues were identified by case-managers or Intervention participants as a problem during at least one telephone discussion by 43% and 36% of participants (*n* = 204), respectively. Analysis of the recorded telephone calls highlighted that case-managers often talked about strategies to treat and prevent pressure injuries. These strategies were not different to what participants had been taught as inpatients at CRP prior to discharge. However, some participants indicated they had forgotten what they had been told to do at CRP, and that the regular telephone calls with their case-managers were helpful reminders. This is a typical comment provided by a participant:

“*If I have any problems related to my health or I see any blackish spots on my back then I share them with him (the case-manager). Then he guides me about how I should take care of my problems. Sometimes he discusses with me in-depth about why I am getting these problems. For example, sitting or lying in the wrong position. He advises me on how I should lie or sit. He teaches me these types of things, so I feel good. I also feel good that I can share my problems with him*.” (Participant with tetraplegia and minimal health problems).

It was not always easy for case-managers to help participants with pressure injuries over the telephone. In particular, case-managers described how sometimes participants under-reported pressure injuries and it was difficult for case-managers to gauge the seriousness of the situation. Case-managers tried to overcome this by encouraging participants, where possible, to send photographs of pressure injuries by smartphone. However, this strategy was not widely implemented because either participants did not have smart phones or it was too costly for them to send images. In addition, case-managers spoke to family members to confirm participants’ reports. Case-managers also discussed how it was impossible for some participants to follow their advice. For example, some participants did not have adequate family support to help lift or turn them regularly. Others were advised to remain on bedrest but were unable to comply because they needed to work to support their families. The case-managers also reported feeling helpless once pressure injuries became severe. In these cases, the case-managers would call upon the expertise of other staff at CRP to guide them and the participants. They also spoke directly to family members to help them with dressings and to provide support. Case-managers often tried to help participants access available local services or gain readmission to CRP but reported that often they could not find appropriate health services to assist or could not get participants readmitted to CRP because of limited bed availability. In some cases, participants were offered a bed at CRP but could not travel the long distance required to get there. Similarly, some participants expressed frustration at the lack of SCI expertise at their local hospitals.

*“I discussed this with the [case-manager]. He told me that if I was unable to come to Dhaka [location of CRP] then I needed to get admitted to a nearby medical centre. So I went to [name withheld] medical centre late one night at 3am … They didn’t know anything … I was admitted to the hospital for ten days but the doctor did not once advise me to change my body position … They don’t have any idea about SCI…”* (Participant with tetraplegia and minimal health problems).

Case-managers stated that some participants were very depressed and lacked hope for the future. They felt that this sometimes prevented participants from heeding the case-managers’ advice and being pro-active in treating and preventing their pressure injuries.

### Theme 2: Participants and the case-managers valued the home visits although they were logistically difficult to conduct

Home visits were highly valued by case-managers. Home visits enabled case-managers to further assess participants’ home environments and equipment (e.g. type of mattress) and identify environmental and structural barriers (e.g. steps). Case-managers also valued the opportunity to directly assess participants (e.g. look at their skin) and to directly provide recommendations to address observed barriers and problems. Home visits also gave case-managers an opportunity to raise awareness in the community about supporting people living with disabilities. This was possible because often community members would be supportive and curious about the home visits, and come to participants’ homes when the case-managers were present. Importantly, some participants stated in the interviews that the home visits made them feel special and cared for.

A case-manager summarised the benefits of the home visits with this statement:

*“…when we visited the patients’ homes, they could see our faces and we could share our facial expressions. Also, when I saw a person’s family condition then I had a better understanding of the problems and more sympathy. Also, during the home visits, the patients started to trust me. As I said earlier, some patients initially hid their problems from me after discharge [when speaking on the telephone]. So, through the home visits I could see the situation and home environment. I tried to modify their home environments.”* (Case-manager)

However, case-managers also noted that the home visits required significant effort, time and organisation. Some home visits required 1–2 days of travel. The travel was particularly challenging for a female case-manager because it is unusual and difficult for women in Bangladesh to travel alone outside their home communities and particularly after sunset. In addition, case-managers stated that sometimes they worried about accidents while travelling because of the poor roads and the need to travel long distances using cars, motorbikes and ferries.

### Theme 3: Telephone calls and home visits helped alleviate a sense of social isolation and depression

All the participants described how the initial few weeks after discharge from CRP were extremely difficult as they tried to adjust to their new lives. The regular telephone calls and home visits provided participants with a link back to the safe and inclusive environment of CRP. Participants described how the telephone calls helped to reduce their sense of social isolation as they valued speaking to people who were comfortable in discussing their problems and who had an in-depth understanding of their challenges. In comparison, some participants described how people in their local communities did not know how to talk to them, and treated them differently than prior to their injuries, and how families and community members became tired of being asked for constant ‘favours’ such as help with transfers or financial assistance. A participant reported:

“*We may not have any communication with anyone other than with them [their case-managers]. This makes us feel good…*” (Participant with tetraplegia and minimal health problems)

Case-managers tried to address social isolation and encourage active community participation where possible. For example, they encouraged participants to go outside their homes and attend their local mosques. This was more feasible for some participants than others because it was dependent on family support and wheelchair access. Some participants described social isolation due to family breakdowns, during which the case-managers would try to intervene and support spouses and family members encouraging them to stay together, if and where appropriate.

### Theme 4: Case-managers inspired trust and confidence, though setting up an action plan with participants was an unfamiliar approach that became more familiar over time

Participants appeared to have trust and confidence in their case-managers which seemed to be facilitated by the rapport built between the case-managers and participants over time, but also through the case-managers’ clinical expertise, and provision of the allocated $AU80 for essential equipment (see Supplementary File 6). Case-managers used an informal and conversational approach to build rapport with participants. For example, the case-managers often started telephone conversations by asking participants if they had eaten, and from there they would do a quick assessment of nutritional intake and provide locally relevant suggestions for dietary changes including vegetables which were currently in season. The case-managers often asked after the wellbeing of family members as a way of gauging whether carers were around, and what supports were available.

A key aspect of the intervention was screening for problems and setting up action plans in collaboration with participants i.e. joint goal setting. This differed from the usual approach in Bangladesh, where health providers typically dictate the actions to be taken in a non-negotiable manner. In contrast, in the CIVIC trial, case-managers were encouraged to facilitate a goal setting process where participants set their own goals, reflected on how to achieve their goals through an action plan, and regularly reviewed progress with case-managers. The goals were diverse and ranged from better skin management to community or family participation. At the beginning of the trial, case-managers had little experience helping people set goals and plans of action. Neither were participants familiar with this approach; as most participants expected case-managers to provide them with information. For this reason, case-managers initially required training in this approach and in general principles of psychology. Case-managers reported feeling more confident with this approach as the trial progressed. They described moving from a “*telling the participants what to do approach*” (based on a provided checklist) to a goal setting approach. Case-managers stated that during the early days of the trial they were frustrated because participants did not listen to or heed their advice. They described how they grew to better understand the many reasons why participants did not always adhere to advice, and they became less critical in the way they interacted with participants. Instead, over time, the case-managers became better at encouraging participants to self-reflect on their problems and possible solutions, and to set goals that took into account competing priorities.

This quote illustrates the reflections of a case-manager on his/her role in facilitating and enabling participants to develop an action plan:

“*Lots of patients have opportunities but there is nobody for them to discuss opportunities with them. Nobody has time to talk with them… We let (the) patients know about the sorts of opportunities they have for their future*.” (Case-manager)

### Theme 5: Limitations of the financial allowance and opportunities for employment

The provision of $AU80 was mainly intended to support the prevention and management of serious complications through the provision of basic equipment (e.g. bladder supplies; see Supplementary File 6). However, it was apparent that while this $AU80 was greatly valued, the participants and their families faced significant financial difficulties, which this amount of money could not relieve. The analysis of our records showed that the mean (SD) amount of the allowance expended per participant was $AU73 ($19). Most of these funds were spent on bladder-related equipment such as catheters, urinal bags and lubricant for self-catheterisations (mean, $AU59 per participant). However, all the participants who were interviewed stated that they needed greater financial assistance. The same comment was repeatedly made by the four healthcare professionals. Financial support was often needed to setup small businesses that could ensure an ongoing source of income. For example, at a visit to a participant’s home, a community leader asked for financial support so that the participant could buy some goats as a source of ongoing income. Other requests included support to procure some initial merchandise so participants could set up small village shops.

The case-managers tried to help participants find work to reduce the financial strain on participants and their families. However, even though most participants had received vocational training at CRP (e.g. training to set up a small business, sew or fix machinery), it was evident that there were limited work opportunities for participants in their home communities, especially for those living in rural areas.

## Discussion

The purpose of this study was to examine how our community-based model of care was administered ‘on the ground’ and to gauge the perceptions of the participants and healthcare professionals to the intervention. Overall our model of care seemed feasible, culturally appropriate and valued by the participants and healthcare professionals. There was a strong focus on the prevention and management of pressure injuries through the telephone calls and home visits. Home visits were logistically difficult but were valued by the case-managers and participants. Ongoing regular contact between case-managers and participants partially alleviated participants’ sense of social isolation and depression. Looking across the themes, participants appeared to invest trust and confidence in case-managers, who were often required to address complex clinical, psychological and social problems.

There are four key implications from our key themes for those considering rolling out our model of community-based care in similar settings: First, our intervention built on the education participants had already received at CRP prior to discharge. Therefore, the advice and education provided over the telephone was not always new for participants. The regular contact with the case-managers did, however, serve to reinforce and remind participants of what they had previously learnt, and encouraged participants to look after themselves. It is possible this intervention might not be helpful in situations in which participants had not received prior education at a specialised SCI centre such as CRP, but it is also possible that the intervention could be of more value in these situations. This is an example of the potential importance of ‘contextualisation’ of the results of randomised controlled trials to local factors that may influence outcomes [22].

The second key implication was the importance of the home visits. Home visits were logistically difficult to conduct. That might not be the same for all LMICs because Bangladesh has a very decentralised population. Most participants lived in rural locations with poor transport infrastructure. The CRP is already working on decentralising their SCI services so that travel to people’s homes is less burdensome for staff. This model—one in which satellite SCI services are attached to a larger centrally located SCI centre—is used elsewhere in the world, including in other LMICs such as Vietnam (personnel communications with staff from Handicap International). In addition, CRP is introducing the use of ‘telehealth’ with greater use of face-to-face interactions. However, decentralisation requires maintenance of a skilled workforce at multiple sites, which can be difficult to achieve when skills, support and training opportunities are concentrated centrally. Many of these issues are discussed in the recent World Health Organization report on rehabilitation in health systems [25].

The third key implication of our findings was the importance of the skills and expertise of case-managers. We used physiotherapists with strong clinical backgrounds in SCI and a good understanding of nursing issues including basic care for the bladder, bowel and skin. They received training from psychologists, nurses and doctors prior to the trial. We initially planned to employ a variety of healthcare professionals as case-managers, but only physiotherapists applied for the positions. This reflects the availability of physiotherapists in Bangladesh. They are generally well trained in SCI and capable of providing general advice for nursing and medical problems. However, despite specific training, the case-managers lacked some of the specialist knowledge and skills of well-trained nurses, psychologists and other healthcare professionals. We see this as an inevitable consequence of limited resources.

A particularly important skill required of case-managers is the ability to support behaviour change. Recent rehabilitation studies have highlighted the value of behaviour change approaches and the use of a behaviour change wheel in the systematic development of complex interventions that require changes in participant behaviour and capability [26]. We tried to encourage a behaviour change approach and goal setting but case-managers were initially unfamiliar with both. They required training and support to be able to facilitate patient centred care that was different to the usual culture of delivering healthcare in Bangladesh. Our impression is that it may be more difficult to implement behaviour change approaches and goal setting in this context as compared to caring for people with SCI in high-income countries. Nonetheless, the case-managers were highly motivated and devoted, and very good at developing rapport, trust and a strong therapeutic relationship with the participants; all of which are key to improving people-centred services [27]. It is not clear how easy it would be to employ staff of a similar calibre if the intervention were to be rolled out on a large scale in Bangladesh and other countries. Clearly, the skills and personal qualities of the case-managers may influence the effectiveness of the intervention.

Lastly, the financial strain on participants and their families was a reoccurring challenge that could limit the effectiveness of support provided by case-managers. In LMICs such as Bangladesh, those with SCI and their families often live in extreme poverty. We found that 65% of patients admitted to CRP in 2011 were living below the poverty line when followed up 6 years later [28], and that 91% of the families of participants in the CIVIC trial were thrown into extreme poverty by the loss of the person’s income [19]. This highlights the pressing need of these people and their families for financial assistance after injury, as was also noted in the 2013 World Health Organisation Report on SCI [8]. Looking ahead, it is likely that wider programs of social support and financial protection will be required alongside the design of innovative culturally appropriate programs such as the CIVIC community-based intervention to sustainably address the needs of this marginalised population [29].

Our study had several important strengths. It was undertaken alongside a rigorously conducted trial with pre-specified trial protocol, statistical analysis plan and process evaluation plan. Importantly, to reduce bias, all but two of the investigators remained blind to the trial results which were expected in March 2020. However, the present study is not without its limitations. First there is the potential for positive reporting bias because MSH and LAH helped design the intervention and MSH worked at CRP for many years. This risk was mitigated by the involvement of HL who is an external evaluator. It was also mitigated by the strong emphasis on researcher reflexivity. Second, the recordings of the telephone calls were conducted later in the trial, by which time case-managers had become more experienced. It would have been helpful to have captured some early telephone interactions. None of the telephone calls were conducted with recently discharged participants whose problems may have been greatest. Lastly, we did not interview all the case-managers or the carers of the participants who had died or had poor outcomes. The later may have provided some helpful insights into what went wrong in those cases. We will address these issues in a post-hoc process evaluation that will triangulate findings of the process evaluation with the primary effectiveness analysis of the trial.

The CIVIC trial aims to reduce the high levels of mortality and serious complications in people from LMICs living with SCI. Irrespective of the final results of the CIVIC trial, we hope this process evaluation will help those working in LMICs to design programs to support people with SCI and other disabilities in the community following discharge.

## Supplementary information

Context of the CIVIC trial

Case Reports Forms and checklist

Interview guide

Coding framework

Illustrative quotes

Allocation of $AU 80

## Data Availability

The authors will consider any reasonable requests to access the data.
