# Supplementary material for: Understanding how a community-based intervention for people with spinal cord injury in Bangladesh was delivered as part of a randomised controlled trial: a process evaluation
Source: Spinal Cord. 2020 Jun 15;58(11):1166–75. doi: 10.1038/s41393-020-0495-6 (PMC7606133; doi:10.1038/s41393-020-0495-6)
Supplement: Supplementary file 1 — Context of the CIVIC trial [file 41393_2020_495_MOESM1_ESM.pdf]

Hueiming Liu, Mohammad Sohrab Hossain, Md. Shofiqul Islam, Md. Akhlasur Rahman, Punam D Costa, Robert D Herbert, Stephen Jan, Ian D Cameron, Stephen Muldoon, Harvinder Singh Chhabra, Richard Lindley, Fin Biering-Sorensen, Stanley Ducharme , Valerie Taylor, Lisa A Harvey, on behalf of the CIVIC Trial Collaboration. **Understanding how a community-based intervention for people with spinal cord injury in Bangladesh was delivered as part of a randomised controlled trial: a process evaluation.** Spinal Cord 2020.

Supplementary file 1: The context of the CIVIC trial and the background to the development of the community-based model of care

Context of the CIVIC trial

The CIVIC trial is based at the Centre for the Rehabilitation of the Paralysed (CRP) in Bangladesh: a large not-for-profit hospital that provides acute and rehabilitation care for people with spinal cord injury (SCI). The CRP admits over 350 people with recent SCI each year making it one of the largest specialised hospitals for people with recently acquired SCI in Asia. The hospital largely serves the poor and disadvantaged with most coming from rural areas in Bangladesh and working as labourers prior to injury [1]. Treatment and rehabilitation are provided at no cost although some patients on higher incomes are asked to contribute to the cost of their care. People with recent SCI admitted to CRP receive standard rehabilitation prior to discharge. This includes training in mobility, bladder and bowel care, vocational training, and guidance on ways to find employment and be independent at home and in the community. They also receive education about strategies to manage health-related problems. All participants are provided with essential equipment including a wheelchair and cushion, and some receive mattresses. There are no equivalent specialised hospitals in Bangladesh although there is a government-funded SCI unit in a tertiary level hospital in Dhaka.

Usual care after discharge from CRP is variable. Data collected as part of the CIVIC trial indicated that all participants (control and intervention) had a median (interquartile range) of 2 (1 to 5) telephone interactions with staff from CRP, and were visited by CRP staff a median (IQR) of 1 (0 to 2) times over the first two years following discharge. Follow up is based on a three-tiered system for prioritising patients that is based on level and completeness of injury only. Whilst this system exists it is yet to be fully implemented. Currently, patients are rung and visited in a more haphazard way than perhaps hoped for because of limited resources and many other issues. Patients are not routinely brought back to CRP for an outpatient follow-up after discharge. They are encouraged at the time of discharge to telephone CRP if they experience problems but they cannot easily visit or get readmitted to CRP because CRP has limited bed capacity, and most patients need to travel between 4 and 20 hours on poor roads to get from their homes to CRP. The CRP does readmit some patients with serious pressure injuries and problems but there is a long waiting list for readmission. Consequently, survival is poor. We have estimated 32% are dead at 5 years [2]. Most people with SCI have access to closer local general hospitals but these are under-resourced and generally do not have staff experienced in the management of people with SCI. In addition, patients are often required to pay for care. Not surprisingly therefore, people with SCI and their families tend to manage pressure injuries and other problems unsupported at home which contributes to the high mortality rates.

Development of the model of community-based care

The model of community-based care tested in the CIVIC trial was developed by the investigators over a number of years and tested in a pilot study of 30 people with recent SCI [3]. A component of the intervention was tested in a clinical trial of 120 people with SCI [4].

The model of care stemmed from a belief that most secondary complications of SCI that cause premature death can be prevented with simple strategies. The intervention is based on models of care provided in high-income countries which typically involves case management with standardised protocols. In our model of care, each participant is assigned a healthcare professional for two years from the time of discharge. The healthcare professional acts as a case-manager and is responsible for telephoning participants every two weeks in the first year and every month in the second year after discharge, as well as conducting three home visits. At each point of contact, the case-manager screens the participant for complications, and provides advice and support. The case-manager encourages the participant to identify goals and to work through solutions for their problems. They also speak to family members and friends as needed. The home visits are important for understanding the participants' home environment and for establishing rapport. They also provide an opportunity to conduct a face-to-face assessment with participants and to show them as well as their families and carers how to manage problems. The intervention focuses on early identification of problems and preventing small problems escalating into large problems. This is particularly important for pressure injuries which can quickly become life-threatening if allowed to progress.

The care package includes an $AU80 allowance for each participant over the 2-year period. This is to cover the cost of essential goods and services such as mattresses, catheters, lubricant for self-catheterisations, dressings for pressure injuries, travel to local hospitals. Case-managers closely supervise how the money is spent. All participants are also provided with a pictorial book that provides guidance on key and common issues they are likely to experience post discharge. The book is illustrated so that it can be used by participants with limited literacy.

Références

1. Hossain MS, Harvey LA, Islam MS, Rahman MA, Liu H, Herbert RD. *et al.* Loss of work-related income impoverishes people with SCI and their families in Bangladesh. *Spinal Cord* 2019.
2. Hossain MS, Harvey LA, Islam MS, Rahman MA, Glinsky JV, Herbert RD. A prediction model to identify people with spinal cord injury who are at high risk of dying within 5 years of discharge from hospital in Bangladesh. *Spinal Cord* 2019; 57: 198–205.
3. Hossain MS, Harvey LA, Rahman MA, Bowden JL, Islam MS, Taylor V. *et al.* A pilot randomised trial of community-based care following discharge from hospital with a recent spinal cord injury in Bangladesh. *Clin Rehabil* 2017; 31: 781-789.
4. Arora M, Harvey LA, Glinsky JV, Chhabra HS, Hossain S, Arumugam N. *et al.* Telephone-based management of pressure ulcers in people with spinal cord injury in low- and middle-income countries: a randomised controlled trial. *Spinal Cord* 2017; 55: 141-147.
