# Supplementary material for: Understanding how a community-based intervention for people with spinal cord injury in Bangladesh was delivered as part of a randomised controlled trial: a process evaluation
Source: Spinal Cord. 2020 Jun 15;58(11):1166–75. doi: 10.1038/s41393-020-0495-6 (PMC7606133; doi:10.1038/s41393-020-0495-6)
Supplement: Supplementary file 2 — Case Reports Forms and checklist [file 41393_2020_495_MOESM2_ESM.pdf]

Hueiming Liu, Mohammad Sohrab Hossain, Md. Shofiqul Islam, Md. Akhlasur Rahman, Punam D Costa, Robert D Herbert, Stephen Jan, Ian D Cameron, Stephen Muldoon, Harvinder Singh Chhabra, Richard Lindley, Fin Biering-Sorensen, Stanley Ducharme , Valerie Taylor, Lisa A Harvey, on behalf of the CIVIC Trial Collaboration. **Understanding how a community-based intervention for people with spinal cord injury in Bangladesh was delivered as part of a randomised controlled trial: a process evaluation.** Spinal Cord 2020.

Supplementary file 2: The Case Report Forms containing the checklist used to guide the phone calls, and home visits, and to record allocation of the $AU80 per participant.

Instructions for recording phone calls and home visits

Purpose:

The purpose of all phone calls and home visits with the experimental participants is to monitor and support them in their communities. Every time staff make contact with an experimental participant, he/she should:

1. Confirm the wellbeing of the participant
2. Identify and discuss any symptoms the participant may be experiencing since the last contact. Give advice and refer participants to local health care services if required.
3. Reinforce self-help strategies to the participant and their families to prevent complications and enhance quality of life.
4. Speak to the family members or community leaders about their role in caring for the participant, and give support where required.
5. Encourage the participant and his/her family to use photographs and videos in your communication to help with gaining information regarding their condition.
6. Organise the next phone call or home visit and provide items of care for the participant if needed (e.g. wound dressings and urinary catheters).

Instructions:

1. Summarise every contact you make with an experimental participant using either the "Recording Sheet for Phone Calls" or the "Recording Sheet for Home Visits".
2. Complete the "Recording Sheet for Details of Problems and Interventions" whenever an issue/problem is identified.
3. Complete the "Sheet for Recording Pressure Ulcers" if a participant has a pressure ulcer.
4. Complete the **"Serious Adverse Events"** form if a participant has or has had a Serious Adverse Event since your late contact with the participant. This includes – serious injury, illness or pressure ulcer that may (or could have been) life-threatening or may result (or could have resulted) in further disablement. All hospitalisations should be recorded as Serious Adverse Events. **Immediately** inform the Site Coordinator who will notify [name removed] or [name removed] (who in turn will send to George India and notify the Principal Investigators).
5. Complete the "**Death Notification**" form if a participant dies (this includes if you for some reason become aware that a control participant has died). **Immediately** inform the Site Coordinator who will notify [name removed] or [name removed] (who in turn will send to George India and notify the Principal Investigators).
6. Add more pages throughout as needed and record the page number in the top right corner. The numbering should be consistent with the section (eg. page 1C, page 2C, page 3C).

Tips for phone calls and home visits

The trial staff should confirm the participant's wellbeing, location and progress since last phone call or home visit.
If the participant has died, go to page 18, otherwise use the following questions as prompts to help you open up a conversation with the participant. Record any problems and actions taken.
Complete a "Serious Adverse Event" form if any serious adverse events (page 15).

General Wellbeing:

1. Has the participant moved places of residence since the last phone call?
2. Has the participant suffered any physical injury since your last contact with him/her?
3. Has the participant accessed any local health services since your last contact with him/her?
4. Has the participant been readmitted to hospital for any reason since your last contact with him/her?
5. Is the participant maintaining an appropriate level of independence?
6. Has the participant been getting out of bed and the house?
7. Are there any new development/opportunities to help the participant return to work?

Presence of Complications

8. Is there any evidence of any pressure ulcers?
9. Has the participant reported any bladder problems (eg. incontinence)
10. Has the participant had any signs of a urinary tract infection (e.g., raised temperature, sweating/shivering, cloudy or malodorous urine, tiredness, increased spasticity)?
11. Has the participant had any bowel problems (eg. bowel accidents, nausea, abdominal pain, abdominal distension, vomiting, or anorexia)?
12. Does the participant show evidence of raised blood pressure or autonomic dysreflexia?
13. Does the participant seem depressed?
14. Has the participant had any problems with sleep, appetite, concentration or mood?
15. Has the participant been suffering from any pain since your last contact with him/her?
16. Has the participant had any other miscellaneous problems?

Equipment:

17. Is the participant having any problems with any of his/her equipment (e.g. wheelchair, mattress or pressure cushions, walking aids, other)?
18. Does the participant require any medication or having any problems with existing medication?
19. Does the participant require any other medical supplies? (eg. dressings, catheters)

Serious Adverse Events:

20. Has the participant experienced a Serious Adverse Event?

Follow-up for trial staff

21. Does the participant require an additional home visit or phone call?
22. Has the next phone call or home visit been scheduled?
23. Is the checklist completed?

Record of Intervention provided to Experimental Participants

Participant number: Participant's initials:

__A

Trial staff's name:

Recording sheet for phone calls

Instructions: complete one column on each phone call. Place a tick (✓) to confirm that the item was discussed; a cross (X) to indicate an item was discussed and a problem/issue identified; a "NA" if not discussed/relevant. Please also record the start and finish time of each call. Always record "yes" or "no" for a Serious Adverse Event.

**DATE:**													
**START TIME:**													
**END TIME:**													

**Participant's well-being:**													
1. Change of home													
2. New physical injury													
3. Accessed health services													
4. Readmit to hospital													
5. Independence													
6. Getting out of bed/house													
7. Working													
**Complications:**													
8. Pressure ulcers													
9. Bladder problems													
10. Urinary tract infection													
11. Bowel problems													
12. Autonomic dysreflexia													
13. Depression													
14. Sleep, appetite, mood													
15. Pain													
16. Other miscellaneous													
**Equipment/medical supplies:**													
17. Equipment													
18. Medication													
19. Other medical supplies													

**20. ANY SERIOUS ADVERSE EVENTS (yes or no)**													

Record of Intervention for Experimental Participants

Participant number: Participant's initials:

Trial staff's name:

__B

Recording sheet for home visits

Instructions: complete one column on each phone call. Place a tick (✓) to confirm that the item was discussed; a cross (X) to indicate an item was discussed and a problem/issue identified; a "NA" if not discussed/relevant. Please also record the start and finish time of each call AND total travel time (hours). Always record "yes" or "no" for a Serious Adverse Event.

**Details of home visit**					
Date conducted home visit					
Time started home visit:					
Time completed home visit:					
Total time at participant's home (hours)					

**Details of travel to and from home visit**					
Date left work place:					
Time left work place					
Date returned workplace					
Time returned work place					
Overnight stay (yes/no)					
Time spent in overnight stay (hrs)					
Total time travelling to and from participant's home (hrs) – excluding time spent in overnight accommodation and excluding time at participant's home					

**Participant's well-being:**					
1. Change of home					
2. New physical injury					
3. Accessed health services					
4. Readmit to hospital					
5. Independence					
6. Getting out of bed/house					
7. Working					
**Complications:**					
8. Pressure ulcers					
9. Bladder problems					
10. Urinary tract infection					
11. Bowel problems					
12. Autonomic dysreflexia					
13. Depression					
14. Sleep, appetite, mood					
15. Pain					
16. Other miscellaneous					
**Equipment/medical supplies:**					
17. Equipment					
18. Medication					
19. Other medical supplies					

**20. ANY SERIOUS ADVERSE EVENTS (yes or no)**					

Record of Intervention for Experimental Participants

Participant number:	Participant's initials:		__C
Trial staff's name:	Date:	Phone / Home visit *(circle one only)*	

Recording sheet for details of problems and interventions

Details of problems and advice provided as part of phone calls or home visits to participants

Instructions: If an issue is identified then record its number (from previous page), details, advice provided and other comments. If an issue is identified that is not on the list (from previous page) and does not have an ID number, then do not provide an ID number but instead describe the issue in a few words. Add more pages as required.

Details of problem:
Advice provided:
Other comments/ follow-up for next call/home visit:

Details of problem:
Advice provided:
Other comments/ follow-up for next call/home visit:

Record of Intervention for Experimental Participants

__C

Participant number: | Participant's initials:

Trial staff's name: | Date: | Phone / Home visit *(circle one only)*

Details of problem:
Advice provided:
Other comments/ follow-up for next call/home visit:

Details of problem:
Advice provided:
Other comments/ follow-up for next call/home visit:

Record of Intervention for Experimental Participants

Participant number: Participant's initials:

Trial staff's name: Date: Phone / Home visit
(circle one only)

__D

Recording sheets for pressure ulcers

Instructions: If the participant has pressure ulcers, please complete this page every time you make contact with the participant (either by phone or home visit). Add more pages as necessary.

1. Where are the pressure ulcers situated?

   ____

2. For the worst: what is the length and width of the pressure area? Length:________________________
   (please provide the length and width measurements in cm) Width:_________________________

3. For the worst: what does the pressure ulcer look like? (e.g. Is it pink and shiny? Does it have beefy red tissue with a shiny moist or granular appearance? Does it have yellow or white tissue clinging to the ulcer in strings or clumps? Is there any evidence of black brown or tan tissue that is stuck to the ulcer? Is there any drainage present after removing any dressings? How much drainage is there? None, light, moderate or heavy?)

   ____

4. For the worst: is the pressure ulcer better, same or worse since last time you spoke or saw the participant?

   better worse same
   (please circle)

Record any advice given:

____

____

____

____

____

DISCRETIONARY MONEY

Purpose:

1. To capture money spend on each experimental participant.
2. These data will form an important part of the economic analysis.
3. These records are important for accountability and providing details to the Australian funders about how money was spent.

Instructions:

1. Each experimental participant has been allocated $AU80 for the 2 years of the trial to cover any costs deemed necessary by the intervention team.
2. Any money spent on an experimental participant must be approved by the Site Coordinator.
3. The money can cover costs associated with medication, wound dressings, transport to hospital, doctor/hospital visits, home modifications, micro loan for setting up a business, training for work, temporary/emergency accommodation or anything essential to quality of life.
4. Everything spent on a participant must be records on the next pages.
5. The site coordinator must sign each expenditure.
6. A receipt number must be attained from CRP and the quoted on the next pages.

Record of Intervention for Experimental Participants

Participant number: Participant's initials:

___E

Trial staff's name:

Recording sheet for money spent on experimental participants

Instructions: complete one row each time an experimental participant is either given money or goods. If the goods are provided by CRP then please estimate a cost to CRP and then ensure that CRP is reimbursed from the project. A receipt needs to be provided from CRP for any money that moves between the project and CRP for goods or services provided to experimental participants.

Date	Details of item that money was spent on	Cost per item	No. of items	Total cost	Method of payment (cash or goods)	Receipt no.	Approved by (signature)
15th Jan 2010<br><br>**EXAMPLE ONLY**	Eg. Catheters<br><br>**EXAMPLE ONLY**	BDT 200<br><br>**EXAMPLE**	2<br><br>**ONLY**	BDT 400	Goods<br><br>**EXAMPLE**	#4052<br><br>**ONLY**	[signature of site coordinator]<br><br>**EXAMPLE ONLY**

Record of Intervention for Experimental Participants

Participant number: Participant's initials:

__E

Trial staff's name:

Date	Details of item that money was spent on	Cost per item	No. of items	Total cost	Method of payment (cash or goods)	Receipt no.	Approved by (signature)
