# Supplementary material for: Understanding how a community-based intervention for people with spinal cord injury in Bangladesh was delivered as part of a randomised controlled trial: a process evaluation
Source: Spinal Cord. 2020 Jun 15;58(11):1166–75. doi: 10.1038/s41393-020-0495-6 (PMC7606133; doi:10.1038/s41393-020-0495-6)
Supplement: Supplementary file 3 — Interview guide [file 41393_2020_495_MOESM3_ESM.pdf]

Hueiming Liu, Mohammad Sohrab Hossain, Md. Shofiqul Islam, Md. Akhlasur Rahman, Punam D Costa, Robert D Herbert, Stephen Jan, Ian D Cameron, Stephen Muldoon, Harvinder Singh Chhabra, Richard Lindley, Fin Biering-Sorensen, Stanley Ducharme , Valerie Taylor, Lisa A Harvey, on behalf of the CIVIC Trial Collaboration. **Understanding how a community-based intervention for people with spinal cord injury in Bangladesh was delivered as part of a randomised controlled trial: a process evaluation.** Spinal Cord 2020.

Supplementary file 3A: The Interview Guide for Trial Staff **(**Source 2)

THE CIVIC TRIAL-PROCESS EVALUATION
RECORD OF INTERVIEW FOR THE TRIAL STAFF

Name of Interviewer: ____

Name of others present: ____

Date: __ __ / __ __ /__ __ __ __
d d / m m / y y y y

PE ID No: ____

Role/Job title: ____

Date of birth: __ __ / __ __ /__ __ __ __
d d / m m / y y y y

Gender: Female / Male

Date commenced in current position: __ __ /__ __ __ __
m m / y y y y

Date graduated __ __ __ __
y y y y

Place of interview: CRP/ Staff members’ home/ Other-please specify ___________

RESEARCHER FIELD NOTES:

How do you think the interview went?

What struck you as important?

What further questions/areas would you like to explore in the next interview?

INTERVIEW GUIDE FOR THE CASE MANGERS

Key questions in bold, with probing questions in non-bold. Of note, the questions do not have to be asked in this order, and not all questions have to be covered.

Say to the staff member: Thank you for taking part in this interview. As discussed, we are trying to find out how we can better care for people with spinal cord injury (and whether the CIVIC intervention was helpful to patients).

We would like to ask you questions some questions but you are not obliged to answer them. However, if you do, please try to answer them as openly and honestly as possible. Your answers will remain confidential.

**MECHANISMS OF IMPACT/CONTEXT: i.e. Exploring health care professionals' perspectives of how, why and for whom the CIVIC intervention did or did not work for?**
**What is your role in the CIVIC trial? Could you describe what your main responsibilities are?**<br>- What have been some challenges?<br>- What have you enjoyed about your role? Could you give me an example?<br>- Compared to when you first started the role, and now - what have you learnt? Has this changed your care of patients with SCI?<br>- What could help you do your job more effectively?<br><br>**What do you think were the key features of the intervention? Which were really important and why? Could you give me some examples? How was most of your time with patients spent?**<br>- Could you tell me more about the 1st Home visit and what you would typically do? Were you surprised about anything when you visited the patient?<br>- What are some of the differences between the home visits and the phone calls? What did you think about the timing and frequency of the home visits and phone calls (dose)? Were they sufficient or too often?<br>- Did you feel that you could provide education on the prevention of pressure ulcers. If so, when you provided education on the prevention of pressure ulcers - were the patients and family carers able to follow your advice? Why or why not?<br>- Could you describe a typical case of how you would screen, advice and monitor for complications? What types of issues would you usually identify? What did you do then?<br>- Did you feel that you could support your patients emotionally and mentally? If so,

could you provide an example of how you supported a patient emotionally and mentally? (probe about relationship/intimacy support)

- Did you fell that you could advocate for greater care for your patients? If so, can you give me an example of how you advocated for greater care for a patient? When did this work or when did it not work?
- CIVIC provided financial support to patients. How were these funds commonly spent? Where there things that you would have liked to have spent money on but could not? Why is that?

Were there certain patients in your experience who would benefit more from the CIVIC intervention than others? Who and why?

- Was it different caring for patients in the rural area versus urban area?
- Was it different caring for patients in with paraplegia versus tetraplegia?
- Why do some patients have less problems post discharge compared to others?
- Why were some patients more able to comply with your advice and suggestions than others?
- Why did some patients die? In hindsight, do you think these deaths could have been prevented?

IMPLEMENTATION: i.e. Was the trial delivered as intended? Contamination? What are the barriers and facilitators to scaling up the intervention in the future?

We noted XYZ (from the chart audit, or the phone calls). What are some reasons for that?

- Was this because of the timing of the calls? New policy etc.?

What have been some barriers and facilitators in the implementation of the CIVIC intervention?

- Were there any difficulties recruiting participants?
- Was there anything we could have done to deliver the intervention better?

As you know we are hoping that the CIVIC intervention will prevent complications and mortality at 2 years. Do you think we will achieve this? Why or why not?

- Could it be due to patient level factors? e.g. unemployment and financial hardship
- Could it be due to organisational level barriers? e.g. lack of primary health providers

- Are there other policy and environmental level barriers? e.g. environment not suitable for people with to get around

If found to be beneficial, - what would be needed to get the CIVIC intervention incorporated into routine care after the trial?

- Would you want or not want to provide the CIVIC intervention in the future? Why or why not? (e.g. travel fun/tedious, too much/too little time with patients?)
- Do you think other hospitals could provide the CIVC intervention? Why or why not?
- Would the CIVIC intervention be suitable for other LMICs with a similar health system as Bangladesh?

Finally, what would be your ideal model of care post discharge for the:

- Prevention of complications
- Furthering rehabilitation outcomes
- Ensuring psychological and emotional health
- Getting patients back to work

What was your experience being involved in research? Would you be happy to be involved in another research project? Why or why not?

CONCLUDING QUESTIONS/STATEMENT

Is there anything else you would like to say that we have not talked about in this interview?

Thank you so much for your time and for sharing your insights.

Supplementary file 3B: The Interview Guide for trial participants (Source 3)

THE CIVIC TRIAL-PROCESS EVALUATION
RECORD OF INTERVIEW FOR TRIAL PARTICIPANTS

Name of Interviewer: ____

Name of others present: ____

Date: __ __ / __ __ /__ __ __ __
d d / m m / y y y y

PE ID No: ____

Participant ID number: ____

Date of birth: __ __ / __ __ /__ __ __ __
d d / m m / y y y y

Date of randomisation: __ __ / __ __ /__ __ __ __
d d / m m / y y y y

Type of Injury: Paraplegia / Tetraplegia

Gender: Female / Male

Place of interview: CRP / Participant's home / Other – please specify

____

RESEARCHER FIELD NOTES:

How do you think the interview went?

What struck you as important?

What further questions/areas would you like to explore in the next interview?

INTERVIEW GUIDE FOR TRIAL PARTICIPANTS

Key questions in bold, with probing questions in non-bold. Of note, the questions do not have to be asked in this order, and not all questions have to be covered.

Say to the participant: Thank you for taking part in this interview. As discussed, we are trying to find out how we can better care for people with spinal cord injury (and whether the CIVIC intervention was helpful to you).

We would like to ask you questions about your injury and how you have been managing. If at any time this is distressing you, and you would like to stop, please do let us know and we can stop the interview.

**CONTEXT: To understand the patient journey and health care experience**
**Can you tell us about your injury?**<br><br>**What care did you receive in the hospital?**<br>- What were some of the good/bad things about the care you received while in hospital?<br><br>**How was the rehabilitation you received while in hospital?**<br>- What were you provided with when you were discharged? (e.g. mattress, or wheelchair, catheter?)<br>- Did you and your family feel like you would be able to cope after discharged home? Why or why not?<br><br>**Could you describe what the first month after discharge was like for you?**<br>- What type of supports did you get to help you? (Probe on family, community, religious, community health services support.)<br>- What did you and your carer do to prevent complications?<br>- Did things get better over time? How and why?<br><br>**Since discharge, what supports/ services have been most important to you and why?**<br>- Can you provide an example of a problem you had and the help you got? (e.g. pressure ulcer)<br>- Are you supported by other patients with SCI? (e.g. through a whatsapp chat?) Would that be helpful?<br>- What services were available that helped you in:<br>o Preventing complications

- Rehabilitation
- Obtaining employment

How is life for you now? What is your day normally like? (e.g. are you working, relationship with family)

- Can you describe your experience in how has it been to get employment?
- How has your family coped with your injury and not working?

Is there something CRP or anyone else could have done to help you after your discharge?

Additional questions <u>only for</u> CIVIC Intervention participants:

Implementation/ mechanisms:

Satisfaction/ problems with the CIVIC intervention package for the intervention group

As you know, we are exploring if the phone calls and home visits you received were helpful. So the next questions will explore the care you received in greater detail. Please feel free to be honest about what it was like for you, as any feedback you provide will help us improve the care we provide.

What has been most helpful?

What was not helpful?

Probe further on:

- Joint goal setting for skin care and prevention of pressure ulcer
- Screening, advice and monitoring for complications
- Psychological support- level of rapport, positive and supported.
- Getting additional services
- Funding for specific things

Can you tell me about the follow up phone calls you received from CIVIC staff?

- How did you feel when a CIVIC staff member first called you?
- What did you talk about?

Can you tell me about the first home visit and how that was like for you? What about the 2nd and 3rd home visit?

- Were the home visits helpful for you? Why or why not? Can you give an example?
- Were you physically assessed and if so, how did you feel about this?
- Did staff show you how to do things and give you information? If so, was this helpful?
- Would you have liked more visits or was twice in the first year and once in the second year okay?

Specific to participants depending on their notes:

We noted in your records, that XYZ was a problem that XXX helped you with.

- Was that useful? Could you give me an example?
- What was helpful? (e.g. joint goal for skin care and prevention of pressure ulcer, advice provided, and screening of complications)
- What was not helpful? Could you describe a time when it was so.

Maintenance: Translation to current practice

Would you recommend this model of care to others? Why or why not?

As you know, this was a trial, and the cost of providing this care was covered by a research grant. Would you be willing to pay for such services in the future?

Is there anything else you would like to share with us that we have not covered?

Thank you for taking part in this study. If you have any further questions, please feel free to contact us.
