# Supplementary material for: Understanding how a community-based intervention for people with spinal cord injury in Bangladesh was delivered as part of a randomised controlled trial: a process evaluation
Source: Spinal Cord. 2020 Jun 15;58(11):1166–75. doi: 10.1038/s41393-020-0495-6 (PMC7606133; doi:10.1038/s41393-020-0495-6)
Supplement: Supplementary file 4 — Coding framework [file 41393_2020_495_MOESM4_ESM.pdf]

Hueiming Liu, Mohammad Sohrab Hossain, Md. Shofiqul Islam, Md. Akhlasur Rahman, Punam D Costa, Robert D Herbert, Stephen Jan, Ian D Cameron, Stephen Muldoon, Harvinder Singh Chhabra, Richard Lindley, Fin Biering-Sorensen, Stanley Ducharme , Valerie Taylor, Lisa A Harvey, on behalf of the CIVIC Trial Collaboration.

Understanding how a community-based intervention for people with spinal cord injury in Bangladesh was delivered as part of a randomised controlled trial: a process evaluation.
Spinal Cord 2020.

Supplementary file 4: The coding framework

**Mechanisms of action of the CIVIC intervention**	**This node contains what we found in the CIVIC phone calls of what the interactions between staff and participants are like, and what was described in the interviews as important mechanisms of the intervention. This includes pre-specified education, screening, psychological support, provision of equipment and provision/referral to health care services; and inductive codes from the interviews and the phone calls. (Sub nodes expanded upon below)**
**Education**	Education provided for the prevention and management of complications. This includes sub nodes of: diet and nutritional advice, following up on previous problems, the use of the information picture book, and educating the carers in what to do.
**Home visit**	Descriptions of the home visits and what is done. This includes sub node: home modifications, providing advice about the environment, and logistical issues related to the home visits.
**Joint goal setting**	CIVIC staff describing goal setting and problem solving with the participants and discussing opportunities such as employment.
**Motivation of participant**	Descriptions of the motivation of the participant which includes both positive examples and a lack of motivation, and reasons.
**Participants not following CIVIC staff advice**	Descriptions of why participants don't follow advice- could be motivation, could be lack of carer support, lack of resources and capacity to afford food etc.
**Phone calls**	Descriptions about the phone calls as part of the CIVIC intervention, and what was done. This includes case managers and participants describing how the phone calls helped in their initial transition to home, how they manage the pressure injuries using the phone calls, and why participants do not follow advice.
**Providing financial support and equipment**	Mattress, catheter, dressing, jelly, medications, usually from the 80 AUD.
**Referral and redirecting**	Includes organising referral.
**Screening of complications**	Excerpts from the phone calls of how this is done, and also as described by the health care providers and participants. This includes sub nodes: questions about pressure injuries, bladder and bowel management, and checking lifting.

**Support**	General descriptions of support from health providers and participants. Includes some specific examples about managing wheelchairs, dietary advice, and psychological support.
**Timely and convenient care**	Descriptions of CIVIC staff providing general health care when there is limited access. This includes sub nodes: advice about medications, addressing sleep disturbances, and how to stay well.
**Trust and relationship between CIVIC staff and participants**	Aspects of the therapeutic relationship. This includes sub nodes: advice, advocacy of the participant, counselling, enabling participants to return to work, encouraging and motivating participants, counselling. (with inductive codes as indicated below).
**Context**	**A description of the contextual factors that would inpact upon the participants' outcomes. These includes the Individual factors (i.e. Descriptions of participant's journey and challenges, and health care), Organisational factors (i.e. Inpatients and outpatient care at CRP, and in health care system in Bangladesh) and Policy Levels (current rehabilitation policies, spinal cord associations)**
**Trial Implementation**	**Key factors to be assessed as part of the process evaluation, which includes the sub nodes: Blinding, Contamination, Barriers and Facilitators, Staff Job**
**Outcomes**	**Key participant outcomes which includes sub nodes : Psychosocial, Pressure injuries, Complications, Mortality**
**Recommendations for future care**	**Suggestions about how to improve care for people with spinal cord injury. This includes suggestions on how to improve CIVIC intervention to general recommendations of policy changes to address accessibility and societal attitudes, to organisational programs such as the use of telemedicine, peer support and financing structures.**
