# Supplementary material for: Understanding how a community-based intervention for people with spinal cord injury in Bangladesh was delivered as part of a randomised controlled trial: a process evaluation
Source: Spinal Cord. 2020 Jun 15;58(11):1166–75. doi: 10.1038/s41393-020-0495-6 (PMC7606133; doi:10.1038/s41393-020-0495-6)
Supplement: Supplementary file 5 — Illustrative quotes [file 41393_2020_495_MOESM5_ESM.pdf]

Hueiming Liu, Mohammad Sohrab Hossain, Md. Shofiqul Islam, Md. Akhlasur Rahman, Punam D Costa, Robert D Herbert, Stephen Jan, Ian D Cameron, Stephen Muldoon, Harvinder Singh Chhabra, Richard Lindley, Fin Biering-Sorensen, Stanley Ducharme , Valerie Taylor, Lisa A Harvey, on behalf of the CIVIC Trial Collaboration. **Understanding how a community-based intervention for people with spinal cord injury in Bangladesh was delivered as part of a randomised controlled trial: a process evaluation.** Spinal Cord 2020.

Supplementary file 5: Illustrative quotes

Theme 1: Prevention and management of pressure injuries was a major focus of the telephone calls

A typical example of a routine telephone calls provided by the case-managers to an Intervention participant (the participant had paraplegia and many health problems):

> *Case-manager: Are you taking care of your skin every day to see whether there is any kind of sore or pressure?*
> *Participant: Yes I check it every day.*
> *Case-manager: What is the condition of your wheelchair? Is it all right?*
> *Participant: Wheelchair is ok but it's shaky. I need to change its bearings.*
> *Case-manager: Where do you get the bearings?*
> *Participant: The road is not so good here so the bearings are damaged. I buy them from Keshobpur. Earlier you gave me one then I got another from Keshobpur.*
> *Case-manager: Ok good. Sometimes it is necessary to change the bearing and use oil. Do you do that?*
> *Participant: Yes, I use this once daily. It helps me to move easily.*
> *Case-manager: Yes, you are very diligent. Be careful this winter and dress warmly otherwise you may become ill. When you use the wheelchair, you should wear gloves to keep your hands warm. They do not cost very much. Only about 200-250 taka. You should also wear socks.*

Although there were participants who had lost hope and motivation to look after themselves, there were some participants who maintained a positive outlook. This is an example of how a participant was encouraged by a fellow patient he met at CRP to stay 'happy' in spite of the difficult circumstances.

> "*I asked him "You are the only earning member of your family, but you are now a spinal cord injury patient, how will your family cope? He replied "... if I get 100 taka per day for shopping, I will be happy. (My family) is Allah's property, Allah will take care." From (his response) I got courage. If he who has 6 daughters and has barely 100 taka to shop at the market, if he can think like this, then Allah can keep me happy too.*"
> (A participant with tetraplegia and minimal health problems)

If more treatment was required for severe cases or other complications, case-managers would advise participants to access medical care:

> **"***So we tell them to visit the doctor and inform me what he advises. Some of my patients visited doctor, because the clinic was accessible and there was someone to carry him, so they visited the doctor and informed me. But for those patients who couldn't get to a doctor, in those cases we get our doctor (here at CRP) to speak to the patient over the phone" (Case-manager)*

Theme 2: Participants and the case-managers valued the home visits although they were logistically difficult to conduct

A case-manager described what is done during the home visits:

> **"***I think home visits are very important and necessary. This is especially so for some patients as they need practical help. Some patients need to stand in their home with the help of some simple equipment. They need to have some gait training, they need to have some sorts of training in the wheelchair or they will develop contractures or problems, and they need to be shown some exercises." (Case-manager)*

A participant reported how the case-manager taught her an alternate way of lifting during a home visit (a participant with tetraplegia and minimal health problems):

> *Participant: Yes, she used to come to see me (at home). She also checked my wheelchair and lift. We did not learn from CRP how to lift in prone. She taught me that in my home ....*
> *Interviewer: She taught you how to lift in an alternative way so that your pressure ulcer could heal?*
> *Participant: Yes*
> *Interviewer: OK, very good.*

Theme 4: Case-managers inspired trust and confidence though setting up an action plan with participants was an unfamiliar approach that became more familiar over time

> **"***It's getting them (case-managers) more confident over time. So, I think over time it's getting better due to their experience and their knowledge…I think there were a lot of situations, (the case-managers) managed very nicely.... For example, one patient is just staying in his room. He cannot go out because nobody can support him. For another patient, his wife has recently divorced him, and his older mother is taking care of him. When our case-managers go to the participants' homes, they can see the participants have serious problems. So, sometimes when they (the participants) don't do (the proposed solution), the case-managers will try to make the participant laugh, and also understand why the participant did not do the solution (e.g. regular lifting)? And the participant would said, "Oh sir, I don't want to do this because I have this problem". Then our case-manager will explain to the participant that "You need to do it because you need it to stay healthy. It is okay if you don't do it this week, things will be okay. Do it in the next week"...It is making the participants understand that we fully support them.*" (A healthcare professional)

Theme 3: Telephone calls and home visits helped alleviate a sense of social isolation and depression

An example of the mutual concern shown during a telephone call.

> *Case-manager: Okay uncle if you have any problem please ring me.*
> *Participant: Okay, I will call you.*
> *Case-manager: Okay*
> *Participant: Keep well*
> *Case-manager: You too, keep well uncle*
> *Participant: Please keep me in your prayers*
> *Case-manager: We will pray for you. Stay well.*" (Participant with tetraplegia and many health problems)

This is an illustrative example of a typical interaction over the telephone whereby a case-manager encourages the participant to look after his mental health:

> *Participant: I am not sleeping. I stay away the whole night and can't sleep.*
> *Case-manager: This is probably because you are staying at home and not doing anything. Your mind is not active.*
> *Participant: Can I take any medications?*
> *Case-manager: No, no you should not take medications… You need to go to the toilet, urinate and change your position at night. If you use sleeping medications, then how will you do these things? Don't you think so? This is why I am asking you to keep your mind active. You need to read or watch television programs such as the sport. Is that okay?*
> *Participant: Yes, I have television, books and everything.*
> *Case-manager: Try to keep your mind active….then you will find that your ulcer will gradually heal.*" (A participant with paraplegia and many health problems)

Theme 5: Limitations of the financial allowance and opportunities for employment

An example of how a participant valued the financial allowance:

> "*Most of the time I do not have anyone to help me. I cannot manage to buy catheters as none are available anywhere. So they (the CIVIC case-managers) bring me catheters because I need them to urinate. If I have any problems (related to my health) I can ask for their advice. This is helpful for me."* (A participant with paraplegia and many health problems)

An example of financial strain:

> *Participant: I gave up running the shop just a few days ago after my baby was born.*
> *Staff: Don't you have anything in your shop to sell?*
> *Participant: No, there is nothing in my shop at the moment*
> *Staff: Okay, why don't you have anything in your shop to sell? Didn't you buy something?*

> *Participant: I had to stop my business for one and half months now because I do not have any money to buy goods to sell*. (A participant with paraplegia and minimal health problems)

This is another example of a participant who was struggling with finances:

> "*For sick people, lack of money is a very serious problem. In every aspect the lack of money creates problems. Because of this situation in our Bangladesh, I remain like this. …. a helpless person who cannot do anything. I can't defecate, urinate or eat by myself. I pray to Allah that he will provide for my wife … I have to think about who is going to provide for my wife and children when I die*." (A participant with tetraplegia and minimal health problems)
