# Supplementary material for: Understanding how a community-based intervention for people with spinal cord injury in Bangladesh was delivered as part of a randomised controlled trial: a process evaluation
Source: Spinal Cord. 2020 Jun 15;58(11):1166–75. doi: 10.1038/s41393-020-0495-6 (PMC7606133; doi:10.1038/s41393-020-0495-6)
Supplement: Supplementary file 6 — Allocation of $AU 80 [file 41393_2020_495_MOESM6_ESM.pdf]

Hueiming Liu, Mohammad Sohrab Hossain, Md. Shofiqul Islam, Md. Akhlasur Rahman, Punam D Costa, Robert D Herbert, Stephen Jan, Ian D Cameron, Stephen Muldoon, Harvinder Singh Chhabra, Richard Lindley, Fin Biering-Sorensen, Stanley Ducharme , Valerie Taylor, Lisa A Harvey, on behalf of the CIVIC Trial Collaboration. **Understanding how a community-based intervention for people with spinal cord injury in Bangladesh was delivered as part of a randomised controlled trial: a process evaluation.** Spinal Cord 2020.

Supplementary file 6: Median (IQR) expenditure of allowance (AUD), for both the whole cohort and for only those on whom funds were expended. Each participant was allocated up to $AU80.

	Median (IQR) cost per participant (calculated for whole cohort) N = 204	Number of participants who received this item	Median (IQR) cost per participant (calculated only for those who received this item)
Bladder supplies	65.1 (52.2 to 76.1)	196	65.6 (55.5 to 76.3)
Wheelchair cushion/mattress	0.0 (0.0 to 0.0)	35	19.4 (5.3 to 21.1)
Exercise equipment	0.0 (0.0 to 0.0)	21	10.6 (7.0 to 14.1)
Medication	0.0 (0.0 to 0.0)	35	3.5 (2.5 to 12.3)
Dressing for wounds	0.0 (0.0 to 1.8)	73	4.8 (1.7 to 12.1)
Commode for toileting	0.0 (0.0 to 1.8)	54	3.5 (3.3 to 4.4)
Walking/standing aids/orthoses	0.0 (0.0 to 0.0)	20	10.7 (8.8 to 28.2)
Miscellaneous	0.0 (0.0 to 0.0)	14	7.5 (2.6 to 16.7)
